# Growth factors that drive aggrecan synthesis in healthy articular cartilage. Role for transforming growth factor-β?

**DOI:** 10.1016/j.ocarto.2024.100459

**Published:** 2024-03-07

**Authors:** Peter M. van der Kraan, Arjan P.M. van Caam, Esmeralda N. Blaney Davidson, Martijn H.J. van den Bosch, Fons A.J. van de Loo

**Affiliations:** Radboudumc, Experimental Rheumatology, Department of Rheumatology, PO Box 9101, 6500 HB Nijmegen, the Netherlands

**Keywords:** Aggrecan, Cartilage, IGF, BMP, TGF-β

## Abstract

**Introduction:**

Articular cartilage makes smooth movement possible and destruction of this tissue leads to loss of joint function. An important biomolecule that determines this function is the large aggregating proteoglycan of cartilage, aggrecan. Aggrecan has a relatively short half-life in cartilage and therefore continuous production of this molecule is essential.

**Methods:**

In this narrative review we discuss what is the role of growth factors in driving the synthesis of aggrecan in articular cartilage. A literature search has been done using the search items; cartilage, aggrecan, explant, Transforming Growth factor-β (TGF-β), Insulin-like Growth Factor (IGF), Bone Morphogenetic Protein (BMP) and the generic term “growth factors”. Focus has been on studies using healthy cartilage and models of cartilage regeneration have been excluded.

**Results:**

In healthy adult articular cartilage IGF is the main factor that drives aggrecan synthesis and maintains adequate levels of production. BMP's and TGF-β have a very limited role but appear to be more important during chondrogenesis and cartilage development. The major role of TGF-β is not stimulation of aggrecan synthesis but maintenance of the differentiated articular cartilage chondrocyte phenotype.

**Conclusion:**

TGF-β is a factor that is generally considered as an important factor in stimulating aggrecan synthesis in cartilage but its role in this might be very restrained in healthy, adult articular cartilage.

## 1 Introduction

Articular cartilage is essential to make smooth movement possible in humans, and destruction of this tissue is typical of diseases like osteoarthritis that lead to loss of joint function. The unique attributes of articular cartilage are the extremely low friction coefficient and the ability to withstand and distribute high mechanical forces. The most important biomolecule that determines the ability to endure compression is the large aggregating proteoglycan of cartilage, aggrecan.

The term aggrecan for the large aggregating proteoglycan of cartilage was first proposed in 1991 [1], but this molecule had been under study for years earlier [2]. Aggrecan is a bulky molecule consisting of a core protein with a large number of glycosaminoglycan side chains that make up a major portion of the molecular weight of this molecule. The aggrecan protein core contains three globular domains (G1, G2 and G3), connected by two linear domains [1]. The interglobular domain joining the N-terminal globular domains G1 and G2 contains keratan sulfate while all of the chondroitin sulfate is attached to the long extended glycosaminoglycan attachment region between G2 and G3. The glycosaminoglycan chains are highly sulfated and incorporation of radioactive (35S)-sulfate by cartilage cells, chondrocytes, is a widely used way to measure aggrecan synthesis in cartilage [3,4]. Aggrecan molecules aggregate to a giant supra-molecular structure by binding of the G1 domain to hyaluronan and this binding is stabilized by a separate globular link protein. The function of the C-terminal G3 domain is not known but it may have properties that contribute to matrix organization [5].

Type II collagen and aggrecan are the main structural biomolecules of articular cartilage. The half-life of type II collagen in cartilage is over 100 years while the half-life of aggrecan is estimated to be days, months or up to a few years, depending on which aggrecan domain is studied, G1 or G3 [3,[6], [7], [8]]. Not all synthesized aggrecan is stably incorporated in the cartilage matrix, depending on the slow maturation of the G1 domain, and the rate of incorporation age-dependently decreases [9,10]. The relatively short half-life, and the not fully efficient incorporation of aggrecan in the cartilage extracellular matrix, requires that aggrecan is synthesized throughout life. This narrative review discusses what are the main drivers of aggrecan synthesis in healthy intact articular cartilage with a special focus on the growth factors Insulin-like Growth Factor (IGF), Bone Morphogenetic Proteins (BMP's) and Transforming Growth factor-β (TGF-β). We restrict this review to healthy intact cartilage since diseased cartilage (osteoarthritis) and isolated chondrocytes respond differently to growth factors than intact cartilage. A striking example is the inhibition of metalloproteinase-3 (MMP-3) expression in bovine explants and stimulation of this in isolated chondrocytes by TGF-β (non-published own data). Studies with intact cartilage (explants) and *in vivo* studies in healthy animals will be discussed. We include studies that do not specify aggrecan but investigate overall proteoglycan synthesis in articular cartilage since this for the vast majority represents aggrecan.

## 2 What drives aggrecan synthesis in healthy, articular cartilage?

It has been shown that aggrecan synthesis in articular cartilage is stimulated via direct and indirect effects of loading but also strongly by growth factors, either systematically or locally produced. With regard to loading, articular cartilage is a tissue that has to be regularly loaded to stay healthy. People with spinal injury lose the cartilage in their knees even more rapidly than that observed in osteoarthritis [11]. Loading affects chondrocyte metabolism amongst others by cellular deformation, receptor activation, altered matrix interactions and the synthesis and activation of anabolic growth factors.

## 3 Measurement of aggrecan synthesis

In cartilage, both *in vivo* and vitro, aggrecan synthesis can be measured by incorporation of 35S-sulfate. This methodology has been used for nearly fifty years [12]. In healthy cartilage, the aggrecan/proteoglycan rate of synthesis is considered to be equivalent with the incorporation of 35S-sulfate in articular cartilage. This is due to the highly sulfated glycosaminoglycan side chains of aggrecan.

### 3.1 *In vitro* aggrecan synthesis

Short term *in vitro* incubation of cartilage explants with 35S-sulfate, maximally a few hours, is a good way to measure the rate of aggrecan synthesis. With longer incubation times one has to take into account loss of newly synthesized 35S-sulfated aggrecan due to proteolytic degradation and diffusion out of the cartilage, amongst others from the cut edges of explants. In general, depletion of 3S-sulfate from the incubation medium does not to have be taken into consideration due to the large surplus in the culture medium compared to the amount incorporated.

Aggrecan glycosaminoglycan synthesis is dependent on the quantity of inorganic sulfate available to chondrocytes. In studies of proteoglycan synthesis *in vitro* one should preferably use physiological medium sulfate concentrations [13]. The various commercially available culture media differ markedly in their inorganic sulfate content. In studies regarding proteoglycan synthesis 'sulfate free' medium is regularly used. However, under 'sulfate free' conditions synthesis is not only decreased but low sulfated glycosaminoglycans were made that were not synthesized during incubation at physiological sulfate concentration. Thus, the use of 'sulfate free' medium should be avoided [14].

### 3.2 *In vivo* aggrecan synthesis

Serum sulfate concentration in different species vary considerably. Human serum has a low sulfate concentration (0.3–0.4 ​mM) while rabbits have one of the highest serum sulfate concentrations (2.0 ​mM) [15,16]. Cows (1.8 ​mM), fetal calves (0.9 ​mM), dogs (1.2–1.8 ​mM) and mice (1.0 ​mM) have intermediate serum sulfate concentrations [16]. The serum sulfate concentrations will affect the specific activity and 35S-sulfate incorporation in aggrecan-related glycosaminoglycans. Furthermore, the half-life of sulfate *in vivo* is several hours, in human about eight, so that after injection of radioactive sulfate incorporation in glycosaminoglycans will rapidly diminish. Autoradiography after intra-venous injection shows that in a normal adult joint incorporation of 35S-sulfate is nearly exclusively in articular cartilage, but in animals with a non-closed growth plate also, age-dependently, in this tissue [17].

#### 3.2.1 Locally and systematically produced growth factors

Growth factors that stimulate aggrecan synthesis in healthy articular cartilage can be either locally produced by the chondrocytes or other joint cells or at a distance and reach the chondrocytes via body fluids. It is demonstrated that a variety of growth factors is expressed by articular chondrocytes and secreted in either an active or inactive form, known examples are IGF [18], Bone morphogenetic proteins (BMPs) [19], Nerve Growth Factor [20,21] and TGF-β [22]. In addition to these, Vascular Endothelial Growth Factor (VEGF), Fibroblast Growth Factor [23], Hepatocyte Growth Factor [24] and Platelet Derived Growth Factor [25] are detected in synovial fluid. PDGF and bFGF can stimulate the synthesis of large proteoglycans (aggrecan) in bovine cartilage explants [26,27] but IGF, BMPs and TGF-β are considered to be the most potent stimulators of aggrecan synthesis in articular cartilage.

Growth factors are produced systemically and locally but these are also stored in the pericellular matrix, the immediate environment of the chondrocyte. The combination of a chondrocyte with its surrounding pericellular matrix is called a chondron. The perricellular matrix has a unique structure and influences chondrocyte function and differentiation [28,29].

The pericellular matrix is a reservoir for growth factors that bind to structural elements and other proteins in this matrix. For instance, FGF-2 is bound to the proteoglycan perlecan and has a function in mechanotransduction [30,31]. Latent-TGF-β is bound to Connective Tissue Growth Factor (CTGF) in the pericellular matrix and controls the activation of TGF-β [32]. The release of matrix bound growth factors is suggested to be a cartilage repair mechanism involving aggrecan and a sodium flux, that is lost in osteoarthritic cartilage [33].

## 4 Insulin-like Growth factors

The Insulin-like Growth factors I and II, previously called “sulfation factor” and “somatomedin”, were originally detected in serum and thought to be produced in the liver stimulated by growth hormone (GH) (reviewed in Ref. [34]). Mounted up evidence has demonstrated that in addition to production in the liver, both IGF-I and IGF-II are most likely produced in all tissues. In rodent tissues expression of IGF-II declines early after birth [35]. Compression has shown to induce IGF-II expression mRNA expression in cartilage explants of one to two weeks old calves [36]. In humans however, IGF-II levels in serum and tissues persist to be high. In a group of patients (n ​= ​18) with various joint diseases the mean IGF-I concentration in serum was 121 ​ng/ml (range 65–190 ​ng/ml) and 87 ​ng/ml (range 47–277 ​ng/ml) in synovial fluid [37]. IGF's are bound to IGF binding proteins of which six are known and in general inhibit the function of IGF but prolong their half-life [38]. In human osteoarthritic cartilage increased expression of IGF binding proteins has been reported [39].

Chondrocytes are reported to produce IGF-I in an autocrine manner [18]. However, adult bovine and rabbit chondrocytes do not respond with elevated 35S-sulfate incorporation when exposed to GH up to 48 ​h, but were highly responsive to human IGF-I [26,27,[40], [41], [42]]. These results suggest that the action of IGF-I on articular chondrocytes is direct and that the effect of GH on these cells is indirect. Although another study confirms these observations but reports that GH in combination with IGF-I acts synergistically on proteoglycan synthesis in articular chondrocytes [43]. As mentioned above, IGF-I can be produced autocrine by chondrocytes but IGF-I in human serum was shown to be the major driver of proteoglycan synthesis in bovine articular cartilage [37]. Pre-incubation of serum with anti-IGF antibodies strongly reduced the stimulating effects of serum indicating that exogenous IGF has a major role in stimulation of proteoglycan synthesis and that IGF in serum is biologically active, although likely bound at least partly to IGF binding proteins [37]. The fact that blocking was not complete indicates the presence of additional proteoglycan synthesis stimulating factors in serum. Incubation of murine patellar cartilage without serum led to the synthesis of proteoglycans with a smaller hydrodynamic volume than those synthesized *ex vivo* which could be circumvented by the addition of IGF-I [44]. Moreover, proteoglycan synthesis was equally stimulated in patellar cartilage from old compared to young mice over a wide range of IGF-1 concentrations [45].

## 5 Bone morphogenetic proteins

Bone morphogenetic proteins (BMPs) were initially purified from demineralized bone and characterized by their ability to induce new bone formation *in vivo* [46]. BMPs represent a large sub-family of proteins structurally related to TGF-β and comprise a family of some 20 homologous, dimeric factors that control the differentiation and activity of a range of cell types, including many outside bone and cartilage. Several BMPs have been reported to be present in human serum, amongst others BMP-2, 4, 5, 6, 7, 9 and 10 [[47], [48], [49], [50], [51], [52]] and BMP-2, 4 and 7 also in synovial fluid [[53], [54], [55], [56]]. The levels detected in body fluids are very low, between 0,01 and 0,4 ​ng/ml, however, circulating levels of BMP9 have been reported to be high (2–12 ​ng/ml) [50].

In human normal knee joint cartilage mRNA expression of BMP-2, 4 and 6 have been detected and expression of BMP-2 protein in bovine cartilage explants from adult cows [57,58]. Expression of BMP-7 was very low to undetectable in bovine explants [59]. It is not known what local BMP concentrations in healthy articular cartilage are. Using immunohistochemistry, Dell’Accio et al. reported about 40% Smad1/5-P (BMP signaling) positive cells in the middle layer of adult human condylar cartilage [60]. We found no phosphorylated Smad-1/5 staining but strong staining for phosphorylated Smad-2/3 (TGF-β signaling in normal human cartilage [61]. In intact bovine cartilage analyzed *ex* vivo very low BMP signaling was measured by Western blotting of phosphorylated Smad1/5 (unpublished data).

Already in 1992 it was found that recombinant BMP2B (BMP4) and purified osteogenin (BMP3) stimulated proteoglycan synthesis in bovine young, adolescent, and adult explant cultures [62]. In contrast Hills et al. found that BMP-2 and BMP-9 enhanced proteoglycan synthesis in bovine explants in young but not in adult cartilage [63]. These results seem in contrast, but not only different BMPs but also different culture periods, 4 compared to 7 days, and most important different age ranges were used in their experiments. The oldest animals in the study of Luyten were 18–24 months and in the study of Hills 6–10 years. Furthermore, in the study of Hills the old animals did not respond to the BMPs but were highly responsive to fetal calf serum, most likely IGF component. In porcine explants from 8 weeks old pigs BMP-7 dose-dependently stimulated proteoglycan synthesis [64]. These data indicate the BMPs can stimulate aggrecan synthesis but that this capacity is diminished upon ageing.

## 6 Transforming Growth factor-β

TGF-β is a member of a protein family that can be found in all multicellular organisms. TGF-βs regulate many key processes in both health and disease, including cell proliferation, tissue formation, and repair and inflammation [65]. In mammalians, three peptides are present TGF-β1, TGF-β2, and TGF-β3. These peptides have a high degree of homology but differ in their tissue expression. TGF-β′s are synthesized as large pro-proteins that have to be activated before receptor binding and signal via a heteromeric complex of transmembrane serine/threonine receptors, so-called type I and type II receptors, and accessory proteins. The canonical TGF-β type I receptor is ALK5 (Activin-like Kinase5) but also other ALKs (ALK1, ALK2) have been shown to be able to propagate the TGF-β signal, signaling via either ALK5 or ALK1/2 has differential effects on gene expression.

TGF-β is extremely difficult to measure reliable in body fluids such as serum. Platelets contain high levels of TGF-β that can be released when obtaining and processing blood. Furthermore, TGF-β is very sticky which can result in loss of protein during handling. Serum levels of total TGF-β1 are quite high and levels from 20 to 50 ​ng/ml have been reported in healthy people [[66], [67], [68]]. Active TGF-β in serum of human controls vary from 0,22 to 5 ​ng/ml [69,70]. Reported levels of active TGF-β in healthy human synovial fluid are very low to absent. No TGF-β1 could be detected in synovial fluid of temporomandibular of healthy human controls [71].

In articular cartilage, TGF-β is produced locally and is stored in a latent form in large quantities in articular cartilage (∼300 ​ng/ml) [72]. Bovine cartilage explants release TGF-β in culture medium but only in a latent form [73]. Compressive loading rapidly activates TGFβ signaling in bovine cartilage explants, most likely by activation of latent TGF-β [74]. However, when cartilage is unloaded TGF-β signaling is lost within hours. This implies that under normal physiological conditions (regular loading) articular cartilage has a basic level of TGFβ signaling, the default mode for articular cartilage, and expression of TGFβ-regulated genes.

**Fig. 1:**
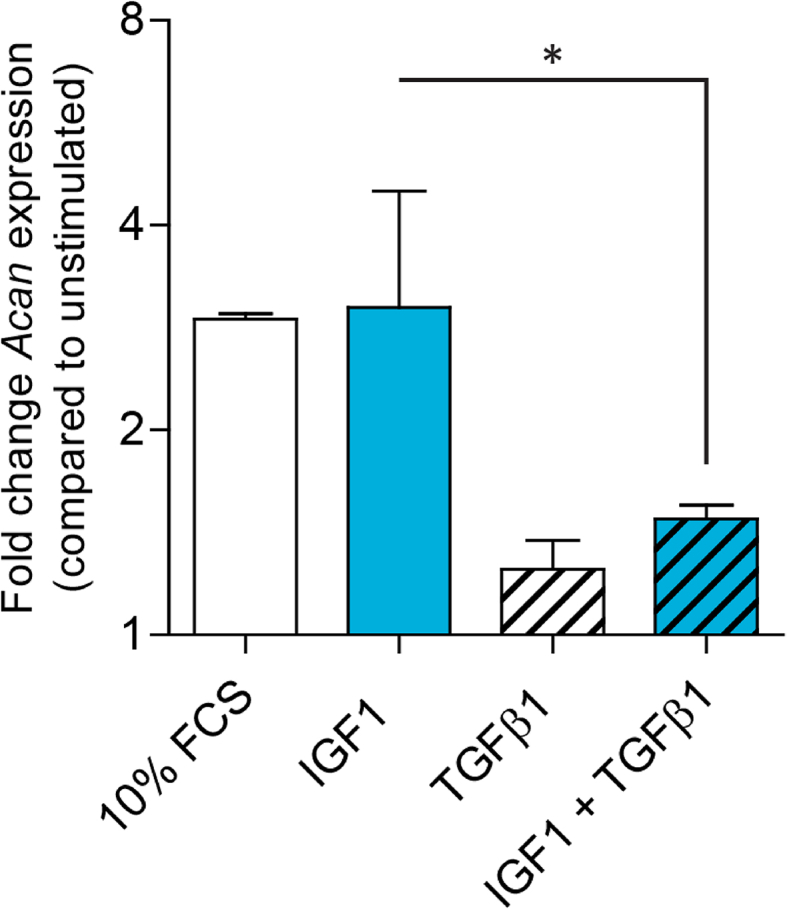
TGF-β inhibits aggrecan mRNA expression under IGF-1 conditions. Aggrecan mRNA (*Acan*) expression was analyzed quantitative RT-QPCR in macroscopic normal metacarpophalangeal cartilage of 4–6 years old cows. Explants were incubated for 2 weeks with 10% fetal calf serum (FCS), 20 ​ng/ml IGF-1 and/or 10 ​ng/ml TGF-β1 (n ​= ​4). Per animal eight explants of 3 ​mm diameter were used, 4 animals were used in total. Medium was partially (50%) refreshed every 3rd day. mRNA was isolated using RNAeasy (Qiagen) and a qPCR was performed using SYBR Green. Statistiscs were calculated using a repeated measures one-way ANOVA in GraphpadPrism (∗ ​= ​p ​< ​0.05).

Morales et al. already reported in 1988 that TGF-β1 and 2 stimulate proteoglycan synthesis in calf (1–6 months) articular cartilage explants [72,75]. Luyten et al. showed that 10 ​ng/ml TGFβ stimulated proteoglycan synthesis in very young calves (1–3 months) 8-fold while this was diminished to 3,5 fold in 18–24 old animals [76]. Cows reach there mature height at an age of 4–5 years and so a young animal of 24 months is still immature and growing. In experiments with 4–6 year old cows we did not find a stimulating effect of TGF-β1 on aggrecan mRNA expression and TGF-β1 even inhibited the stimulatory effect of IGF-I on expression (Fig. 1). Equine explants do show an age-related decline in responsiveness to TGF-β [77] and incubation of fully intact patellar cartilage of 12 week old mice with TGF-β1, up to 5 ​ng/ml, had no significant effect on proteoglycan synthesis [78]. Neutralization of endogenously produced TGF beta with a specific monoclonal antibody to TGF- β1, however, led to stimulation of proteoglycan synthesis, indicating that TGF-β itself inhibits proteoglycan synthesis in anatomically intact murine cartilage. In an additional murine study, in the presence of IGF-I incubation with TGF-β1 resulted in significant inhibition of proteoglycan synthesis [79]. An important study of Hickery et al. using macroscopically intact human femoral condyle cartilage demonstrated that TGF-β1 stimulated proteoglycan synthesis in cartilage of individuals younger than 20 years but not in older ones [80].

In murine *in vivo* studies it was found that a single intra-articular injection of 20 or 200 ​ng TGF-β1 in the knee joints resulted in a slow elevation of proteoglycan in patellar cartilage, only significant after day 3 (30 and 50% enhanced). In contrast, injection of BMP-2 (20, 200 ​ng) led already on day 1 to significantly elevated proteoglycan synthesis with an increase of 275% already on day 2 [81]. These results show that *in vivo* BMP-2 leads to a fast response of articular chondrocytes by heightened proteoglycan synthesis while after TGF-β1 the chondrocytes only slowly react, indicating that this is a secondary but not a direct response to TGF-β1 on proteoglycan synthesis. In summary, these findings imply that TGF-β1 directly stimulates proteoglycan synthesis in immature cartilage but loses this ability in healthy adult articular cartilage.

## 7 Discussion and conclusion

Aggrecan synthesis is stimulated by growth factors but also factors that directly affect chondrocyte function, for instance mechanical stimulation, could play a role. However, numerous studies indicate that added IGF-I, or IGF present in serum, is sufficient to maintain aggrecan synthesis in healthy articular cartilage. The optimal IGF-I concentration to stimulate aggrecan synthesis in bovine cartilage is 20 ​ng/ml [37], concentrations that are found in 20% (human) serum and synovial fluid. However, due to the presence of IGF binding proteins the exact availability of IGF-I in body fluids is unclear. Notwithstanding these uncertainties, it has been shown that in fetal calf serum IGF-I is the main protein that stimulates aggrecan synthesis in bovine cartilage [37,42]. Very few studies have compared aggrecan synthesis *ex vivo*, directly after isolation, with synthesis after *in vitro* culture but we have shown using intact murine articular cartilage that 25 ​ng/ml IGF-I, like 100% mouse serum, can fully maintain the normal level of aggrecan synthesis. In contrast, without IGF-I this drops 50% within 24 ​h [79,82]. In conclusion, IGF-I is the most prominent factor driving aggrecan synthesis in healthy articular cartilage and this factor is responsible for the majority of stimulation of aggrecan synthesis *in vivo*.

The concentrations of most BMPs in body fluids (0,01 to 0,4 ​ng/ml) are far below the concentrations that have been reported to stimulate aggrecan/proteoglycan synthesis in articular cartilage (>10 ​ng/ml). An exception for this is BMP9, levels up to 12 ​ng/ml have been detected in human serum and BMP9 is already active at very low concentrations since it's activity is not blocked by the general BMP inhibitor noggin [50,83]. BMP9 signals via the ALK1 receptor, whether BMP9 will activate articular chondrocytes is therefore dependent on the expression of ALK1 by these chondrocytes. Taking into account the dominant role of IGF-I in driving aggrecan synthesis, the role of BMP9 in this can be anticipated to be small. Furthermore, BMP9 stimulates chondrocyte hypertrophy, a characteristic of osteoarthritis, so high activity of BMP9, or other locally produced BMPs in adult healthy articular cartilage will be an unwanted one [84].

It is a common opinion that TGF-β stimulates aggrecan/proteoglycan synthesis in articular cartilage but this appears to be an oversimplification. It is clear that under specific conditions TGF-β greatly enhances aggrecan synthesis. This is particular true in the process of chondrogenesis both *in vivo* and *in vitro* [81,85,86]. TGF-β is a potent factor in stimulating precursor cells to acquire a chondrocyte phenotype. This TGF-β-induced differentiation is accompanied with a strong increase in aggrecan expression. Furthermore, in isolated chondrocytes and damaged cartilage TGF-β can also drive aggrecan synthesis.

In immature articular cartilage TGF-β stimulates aggrecan synthesis, a function that appears to be lost in healthy adult cartilage. As discussed above, in the presence of IGF, TGF-β can even inhibit aggrecan expression and synthesis. IGF-1 has been shown to stimulate aggrecan synthesis in chondrocytes by the PI3K/Akt pathway [87]. The inhibitory effect of TGF-β on IGF-stimulated aggrecan synthesis might be explained by the blocking effect of the TGF-β signaling molecules Smad2 and 3 on this pathway [88].

**Fig. 2:**
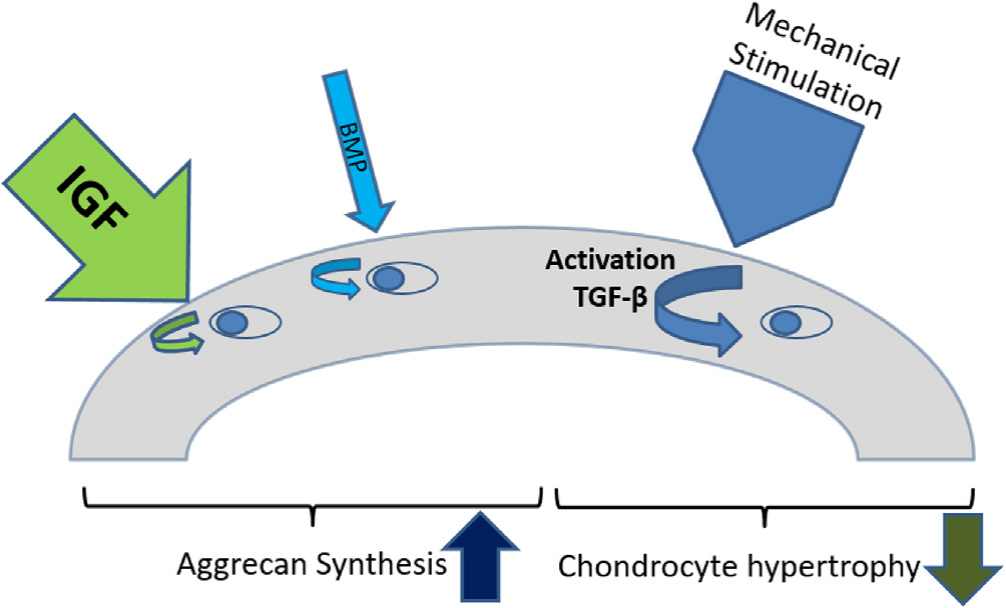
Postulated scheme of the regulation of aggrecan synthesis and chondrocyte phenotype in healthy adult articular cartilage by growth factors. Stimulation of aggrecan synthesis in adult articular cartilage is mainly driven by systemically produced IGF. A small part can be the result of stimulation by systemic BMPs (BMP9) or endogenously produced growth factors. Chondrocyte hypertrophy is inhibited by mechanically-activation of locally present latent TGF-β.

In conclusion, in healthy adult articular cartilage IGF is the main, and needed, factor that drives aggrecan synthesis and maintains adequate levels of production (Fig. 2). BMP's (BMP9) and TGF-β have a very limited role but seem to be more important in this regard during chondrogenesis and in developing and immature cartilage. These factors might further play a role in the cartilage repair response, amongst other by elevated expression and synthesis of aggrecan, that is observed in early (experimental) osteoarthritis [89,90]. The major role of TGF-β is not stimulation of aggrecan synthesis but maintenance of the differentiated articular cartilage chondrocyte phenotype and the inhibition of chondrocyte hypertrophy [74]. So we postulate that in adult normal, healthy articular cartilage IGF drives aggrecan synthesis, BMPs might contribute to this for a small part, and locally mechanically-activated TGF-β inhibits unwanted chondrocyte hypertrophy.

## Author contributions

All authors have made substantial contributions the conception and design of the study, data acquisition, revising the article and final approval.

## Conflicts of interest

No competing interests for any of the authors.
